# How practice in plant collection influences interactions with illustrations and written texts on local plants? A case study from Daghestan, North Caucasus

**DOI:** 10.1186/s13002-020-00376-2

**Published:** 2020-06-23

**Authors:** Iwona Kaliszewska, Iwa Kołodziejska

**Affiliations:** Institute of Ethnology and Cultural Anthropology, University of Warsaw, ul. Żurawia 4, 00-503 Warsaw, Poland

**Keywords:** Daghestan, North Caucasus, Knowledge-making, LEK, Textual plant knowledge, Illustrations

## Abstract

**Background:**

It is only recently that written sources of local knowledge on plants are not being ignored by scholars as not belonging to “traditional” knowledge. Ethnobotanical texts, however, if they at all focus on knowledge from written sources, hardly ever pay any attention to the actual processes of interaction with written texts and illustrations. During our research, we examined people’s interactions with texts, illustrations, and herbarium specimens of plants they collect or are familiar with. We focused on a small community of Shiri people in the mountainous village and in the lowland settlements in the Republic of Daghestan, Russia. In the paper, we address the following questions: how do Shiri people interact with illustrations, written text, and herbaria specimens? How is this interaction influenced by the practice of plant collection? What are the methodological implications of the ways people interact with illustrations, texts, and herbaria specimens?

**Methods:**

Our research was based on long-term ethnographic fieldwork: co-designing of a booklet showing edible plants people collect in Shiri, semi-structured interviews, and video-recordings, and observing interactions between people and text/illustrations/voucher specimens.

**Results:**

We identified three kinds of interactions between individuals and text/illustrations: “text-wayfaring”—predominantly a bodily interaction between an individual and illustrations and text; “fact/spelling checking”—predominantly discursive and information focused; “between wayfaring and fact-checking”—the mix of the two. Using the idea of textual poaching, as well as the knowledge-making approach, we show that the mode of interaction with text/illustrations influences what is acquired, and how. This process influences readers’ LEK. The mere presence of an information in the text available to people does not imply that they will acquire it, make use of it, and change their LEK. Photographs and pressed specimens of locally known plants are often not (or only partly) recognized by the interlocutors. Video-recording is essential for analyzing the above mentioned interactions.

**Conclusions:**

In ethnobotanical research, it is important to pay more attention to people’s interaction with their sources of knowledge, including text and illustrations. The discursive part of LEK is more easily influenced by written sources. The practice of plant collection is not as easily influenced. Ethnobotanists function in a particular context and are embedded in discourses oriented towards conservation of bio-cultural diversity that value heritage as such, so it is important to be aware of one’s positionality. A methodology that relies on showing pressed specimens or photographs to interlocutors may be a very misleading way of collecting ethnobotanical data.

## Background

Written sources of local knowledge on plants were often ignored by scholars as not belonging to LEK (Local Environmental Knowledge) or TEK (Traditional Environmental Knowledge)^1^. On the one hand, scholars as far back as the 19th century realized that there is an influence of written sources on “traditional knowledge” [3]. On the other hand, many ethnobotanists who work in communities with little access to written sources, still try to separate the knowledge from written sources from “pure” TK ([4, 5], and critique of this position by Vermeylen et al. [6]). Such a practice may be, of course, goal determined. If the goal is to look for new medicines, then it might be legitimate to try to separate “pure” TEK. If we want to better understand the processes of knowledge-making, then, we argue, we cannot ignore written sources on plants if these written sources are important to people. Some ethnobotanists (e.g., [5, 7–11]) have already paid attention to knowledge from written sources and showed their importance for LEK of various communities. They, however, did not scrutinize the actual processes of interaction with written texts.

**Fig. 1:**
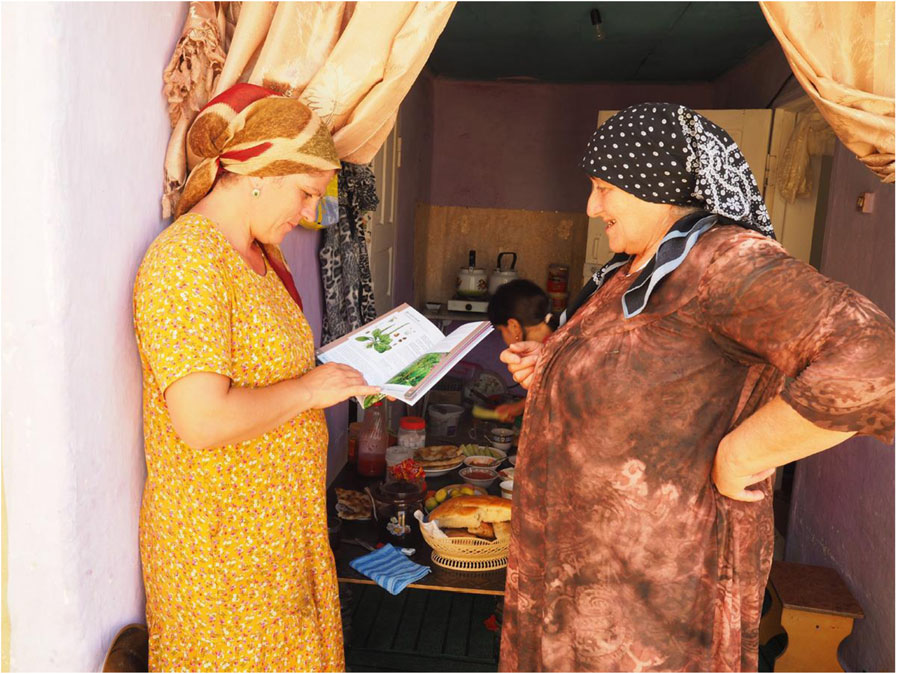
Showing the booklet. Patimat showing the booklet to her daughter-in-law. The photo was taken in September 2017 in Druzhba, Daghestan, Russian Federation. Author: Iwona Kaliszewska

During our research, we examined the interaction of people with texts and illustrations of plants they collect or are familiar with. We focused on a small community of Shiri people living in the mountainous Shiri village and in the lowland settlements and towns (Druzhba, Izberbash, Makhachkala) situated along the Caspian coast in Daghestan. Along with the people from the community, we collected the edible plant specimens [12], then co-designed a booklet containing the local edible plants, and later 2017–2019 distributed it among Shiri dwellers and people originating from the village but currently living in the lowlands (Fig. 1). We filmed the interaction with the booklet.

Daghestan is Russia’s southernmost and the most ethnically and linguistically diverse republic, a considerable part of which belongs to the Caucasus Biodiversity HotSpot. Most Daghestanis are Sunni Muslims. The Shiri village is located in the mountains in the Dakhadaevsky region. People in Shiri speak a distinct language that belongs to the Kubachi subgroup of Dargi languages, and it has not been documented until recently^2^. During the course of our research, the number of households in the village decreased by half and currently only 6 remain. Nowadays, elders from Shiri are encouraged by their children to join them in the lowlands, while the younger generation looks for permanent or seasonal work outside the republic.

Edible plants are important for the elderly and middle-aged Shiri people due to their taste values and their role in forming identity and social relations (for more see [12]), rather than economic reasons. Although plant collection is a predominantly female domain cf. [14, 15], knowledge about wild leafy vegetables eaten on the spot (as snacks) is also shared among boys who, more often than girls, are sent up to herd cattle. Shiri people, both women and men, often share plants or discuss their properties among themselves. Their immense knowledge about wild plants is, however, only partly passed down to the younger generation which lives in the lowlands and either grows their own plants in the garden, or buys them. They visit Shiri only in the summer, and even if they take occasional forest walks during their brief stay, they do not fully engage in plant collection.

Reciprocity and engaging with the community resulted in the booklets brought back to the community. Bringing written materials to the researched community may raise controversies. One of such controversies derives from the idea that TEK is seen as based only on the knowledge transmitted from one generation to the next (cf. [6]). In those communities which have had extensive contact with written sources, the mixing of various sources of knowledge is obvious (global interconnections make it even more visible [5, 16]). The second controversy regarding bringing written materials to the community comes from the idea that the researcher should avoid influencing the community (if it is even possible). In anthropology we do not see it as feasible, realistic, or even desired (cf. [17]). The third controversy concerns standardizing the knowledge [18, 19].

In our opinion, what makes knowledge “traditional” is not the familiarity with particular specimens or the facts about them, neither is the source of this knowledge (e.g., older generations), but it is the fact that this knowledge is made and re-made, and that it is important for the community^3^. The content of local knowledge transforms as a response to changes in the ways of life and biodiversity. This may be seen as an adaptive process (cf. [21]), and that is why we decided to co-design the booklets and distribute them in the community. The *emic* perception of knowledge as being traditional should be taken into account (cf. [20]). It was ethically important to us not to impose the existing categorizations of knowledge quality (with “traditional” seen as “better” by those who see it as “pure” and nature bonded, or as less valuable by others [5]).

It is also important to mention that we had other goals than for example [4] (and a few other ethnobotanical texts on written sources), where the main goals of ethnobotanical and ethnopharmacological research were “input to drug discovery, “conservation of cultural heritage,” and “validation of local pharmacopoeias.” We were interested in the processes of knowledge-making, in particular those connected to written sources and illustrations, and we did not disengage knowledge from written sources from LEK. We follow de Certeau’s idea that reading the text does not necessarily change the person reading it and affect her or his knowledge [22]. The influence is bidirectional. The reader changes the read text as well.

During our research, we tried to answer the following questions: how do Shiri people interact with illustrations, written text, and herbaria specimens? How does the amount of practice in plant collection influence reading about plants and the perception of illustrations? What are the methodological implications of the ways people interact with illustrations, texts, and herbaria specimens? We will address the above questions basing on the process of interaction with written text and illustrations, and the process of co-designing the booklet with Shiri people.

## Methodology

The article is based on the fieldwork among Shiri people in Daghestan conducted between 2015 and 2019^4^. In that period, we conducted four field trips, altogether 12 weeks of fieldwork. Edible plants were collected with Shiri people in 2012–2014 (voucher specimens were deposited in the herbarium of the University of Warsaw Botanic Garden)^5^, then during field trips in 2015 and 2016 we co-designed with Shiri people the booklets with these plants^6^. Eventually, between 2017 and 2019, we distributed the booklets in Shiri and in the lowland settlements (Druzhba, Izberbash, Makhachkala) where people of Shiri origin live. We video-recorded the interaction with the booklets. The idea to create a booklet came in 2015 from Ibragim, the local teacher in Shiri, whose interest in plants had sparked shortly before then, as a result of his son’s illness (cf. [25]). Ibragim wanted to know if plants that grow in Shiri have any healing properties. He also wanted to use such a booklet to teach his pupils about Shiri’s flora. This idea was supported by the school director, Akhmed, and a university teacher, Abdulkadyr. They wanted to have a school/encyclopedia-type booklet with detailed information on plants and their medical properties. It was also important for them to preserve local plant names for future generations. The women did not want to participate in the co-designing of the booklet, claiming that we (the researchers) and the men “would know better, anyway.” Suggestions to include the local usage of plants, or recipes for dishes with those plants, were not really cherished. “What for? Everybody knows it,” commented Ibragim. It seems that the Shiri people wanted the booklet to include the information they did not know or were bound to forget, rather than the everyday knowledge that was in active use. Eventually, the booklet included a botanical description of 27 important edible plants, their habitat and range, medical properties, and a short paragraph on their usage in Shiri. We distributed the booklets to all households in Shiri. In the lowlands, we tried to meet all of our previous informants who migrated from Shiri, as well as some of their relatives. We video-recorded the browsing, reading, or commenting on the booklet in order to grasp the details of the interaction with both the written text and the illustrations.

**Fig. 2:**
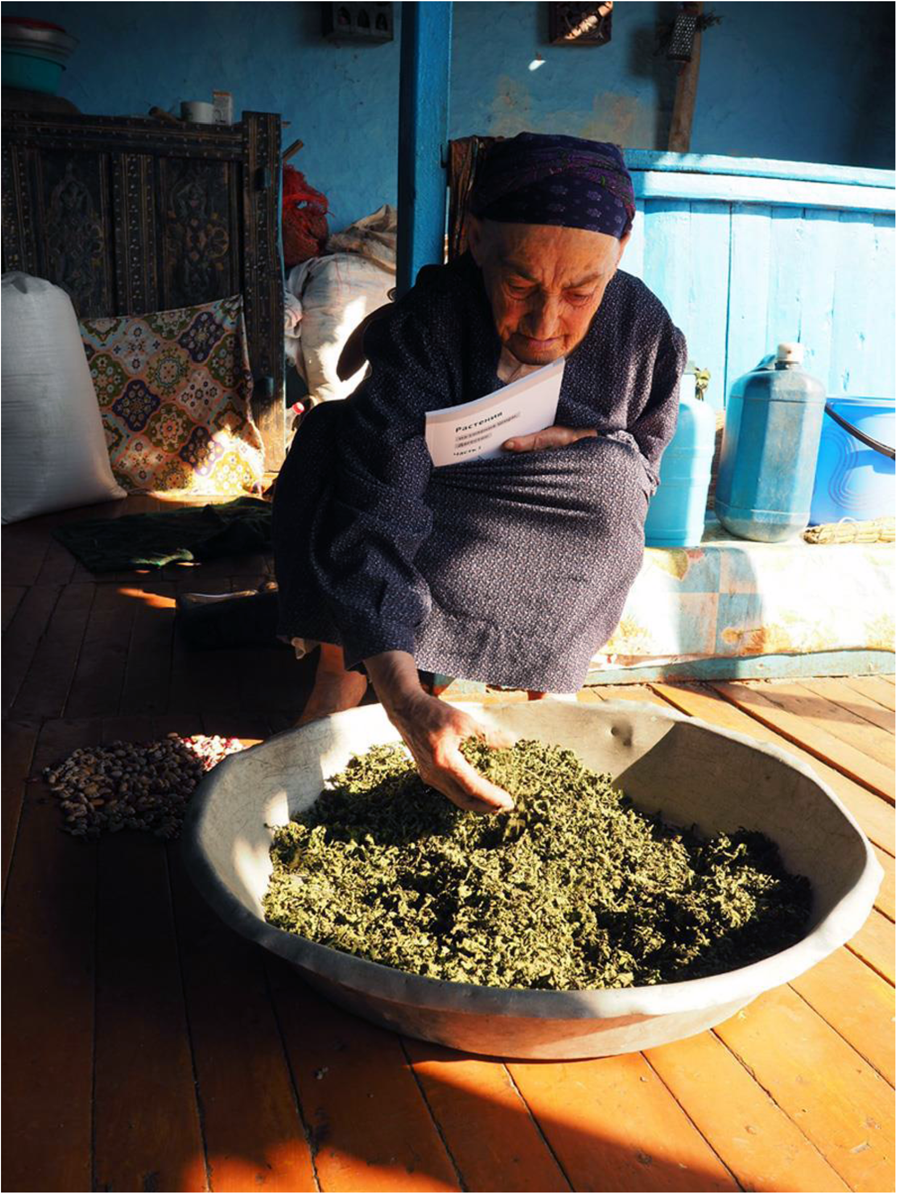
Receiving the booklet. Hadijat, who has a vast amount of knowledge in plant collection was happy to receive the booklet but was not interested in the content. The photo was taken in September 2017 in Shiri village, Daghestan, Russian Federation. Author: Iwona Kaliszewska

We video or audio recorded semi-structured interviews and unstructured conversations with 21 adult women and 13 men originating from Shiri Village and living either in Shiri, or in the lowland settlements of Druzhba, Izberbash, Chinar, or Makhachkala. By the time of that research, our interlocutors were already accustomed to us (and other project members) using small cameras placed on a table or a tripod, so it did not intimidate them. We also noted occasional comments from the other 31 individuals. All of our informants were non-specialists in plant knowledge. The age range was from mid 30s to 90s. Most of them, therefore, received their (at least) elementary education still in the Soviet times (a fact important for the knowledge-making). It is worth mentioning here that, as of 2018, we knew all the Shiri people very well, which allowed great levels of trust and naturalness during the video-recording of their interactions with the booklets, with each other, and with us. In this respect, our methodology was quite specific and, we believe, cannot be randomly applied in other contexts.

Expression of consent was required before each interview and it was obtained verbally. The main language of research was Russian^7^ which is the *lingua-franca* in Daghestan. The older inhabitants used the mix of Russian and Shiri^8^. Russian is the most common language in the lowland settlements, while Shiri is spoken only in the village and in the households of the Shiri people who migrated to the lowlands. In these multi-ethnic settlements, however, most people are fluent in Russian, and it is frequently used also in Shiri households.

## Results and discussion

First, we will present the results and discussion regarding types of interaction with text and illustrations, then we will present methodological implications.

Knowledge about plants was important for all of our interlocutors, even those who have little practice in plant collection. Local plants were a part of their identity and constituted an important part of social relations [12]. Basing on the process of interaction with written text and illustrations, and the process of co-designing the booklet with Shiri people, we identified three kinds of interactions between individuals and text/illustrations/herbaria specimens. The first kind of interaction, “text-wayfaring,” is predominantly a bodily interaction between the individual and illustrations (with or without an additional notice of plant names). It may include tracking the contour of leaves, or of the entire plant. The second kind of interaction, “fact/spelling checking,” is predominantly discursive and information-focused. The third kind of interaction, “between wayfaring and fact checking,” is the mix of the two.

**Fig. 3:**
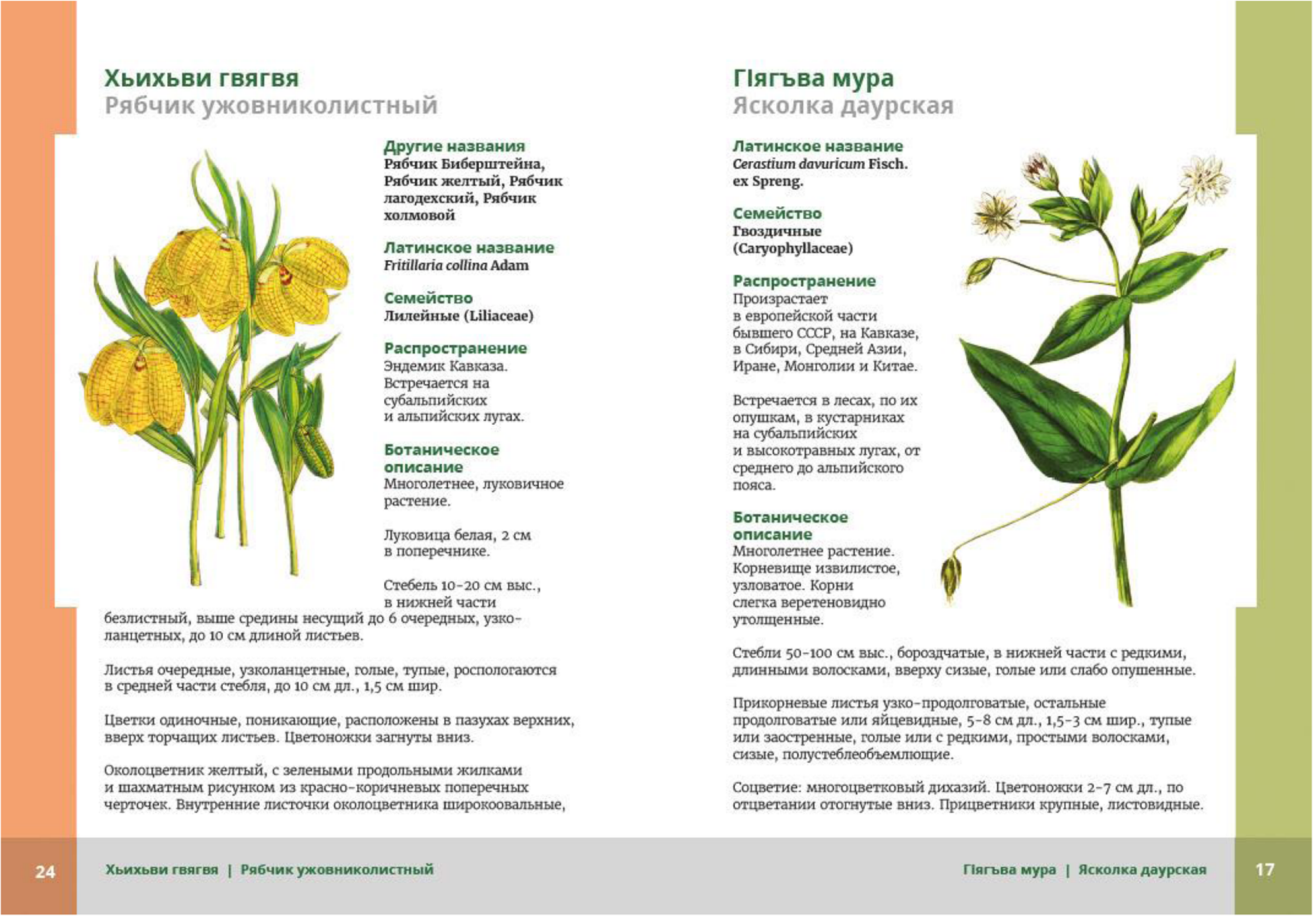
The “proper” booklet entries. Illustrations of plants that were close to 1:1 or very characteristic were considered nice and proper. On the left *kʷakʷi gʷa̰gʷa̰* (*Fritillaria collina* Adam), on the right *ʡaˤʁʷamura* (*Cerastium davuricum* Fisch. ex Spreng)

Patimat (in her 70s) and Malaykat (in her 40s), who had vast knowledge on plant collection, were happy to receive the booklet in 2017, but they did not read it; they just browsed through it looking at the illustrations and plant names (cf. Fig. 2). They briefly looked at the photos/illustrations, and only stopped at those that were close to 1:1 scale, such as *duc’armura* (*Bunias orientalis* L.), *ʡaˤʁʷamura* (*Cerastium davuricum* Fisch. ex Spreng.), *guržinakːʷi* (*Oberna multifida* (Adams) Ikonn.) (cf. Fig. 3). They looked at them with some suspicion, tracing the contours of leaves, or the contour of drawings, as if trying to determine if it was a proper representation or not. It seemed to us that by delineating the borders of the leaf blade they tried to make a visual experience more embodied (bodily connected to the movement). They skipped the close-ups without comment. When we asked them later about these illustrations, they doubted whether this plant actually grows in Shiri, or if this was a correct illustration of the species. When tracing the contour of the drawn plant (for example, in *sːisːupi*, *Allium victorialis* L., and *dagala qʼar*, *Plantago major* L.), they also made a “cutting” gesture when they came to the root which, when asked later, they considered unnecessary/not-useful. Compared to our other interlocutors, they put the booklet away relatively quickly. They were surprised when they heard us discuss the section “Uses in Shiri” with the men present in the room. Three of our interlocutors commented: “we know this anyway.”

Abdulkadyr was then a university teacher in his 60s^9^. When he first received the printed version of the booklet in 2017, he started reading from the table of contents. He right away spotted two spelling mistakes and one typo in the plant names, pointing them to us in a didactic manner (our gender might have encouraged him to assume the authoritative perspective). He took us to his brother, Timur (in his 40s), on the outskirts of Makhachkala. Their family left Shiri when they were children, and now they would visit the village only once or twice a year for religious holidays. Timur’s wife Fatima (in her 40s) lived in Shiri until getting married and often visited the village in summer to collect plants with her mother. The whole family gathered to take a look at the booklet.

**Fig. 4:**
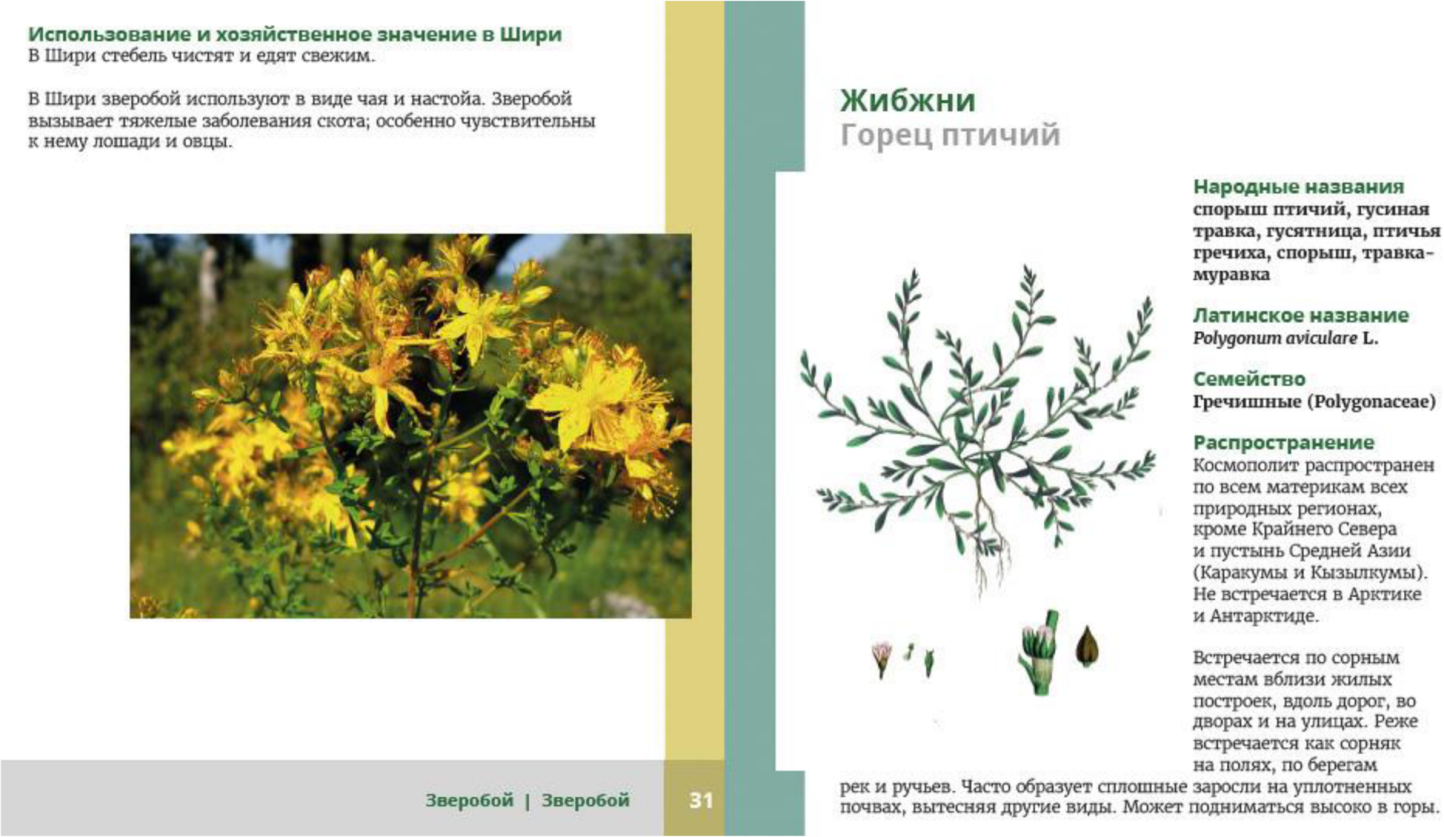
The “improper” booklet entries. Close-ups were usually not recognized, for ex. *zveroboy* (*Hypericum* spp.) on the left. Illustrations with plants abstracted from the environment for ex. *žibžni* (*Polygonum aviculare* agg.) on the right were viewed as wrong

**Fig. 5:**
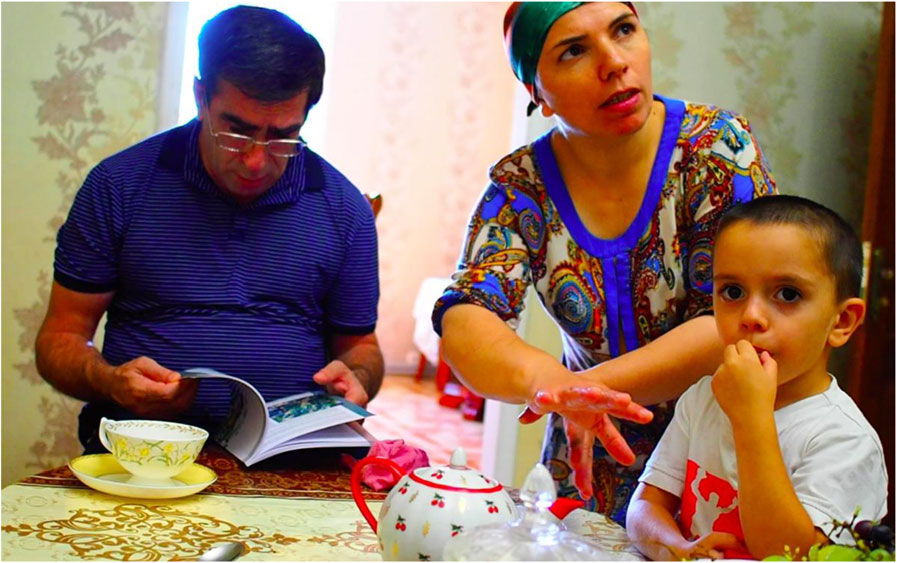
Browsing the booklet. Fatima explains with gestures how *žibžni* (*Polygonum aviculare* agg.) creeps on the ground, her husband consults the booklet with her. The photo was taken in August 2017 in Makhachkala, Daghestan, Russian Federation. Author: Iwona Kaliszewska

“*Žibžni*”—reads Fatima looking at the heading at the top of the page with *Polygonum aviculare* agg. “This is certainly not *žibžni. Žibžni* does not grow up”—she points up (Fig. 4). “It goes like this”—she makes a movement with both of her arms and palms above the surface of the table, to show how the plant grows on the ground and how it spreads (Fig. 5). “It cannot be *žibžni*”—she continues. “*Žibžni* grows in the middle of the village”—she repeats the movement. Abdulkadyr is sitting nearby but not really paying attention to Fatima.

“*Sporish*”—he says looking at the section with local Russian names of *Polygonum aviculare* agg*.* “It really totally cleanses the body^10^, if you drink tea made of it, for half a year, it will truly cleanse your body. A very useful thing,”—he adds.

“Is *žibžni* the same as *sporish*?”—Iwona asks.

“No, *gorets ptichiy* is *sporysh*, it is written here”—he points to the *sporish* name in the local plant names section in the booklet.

“It grows in the centre of the village. It spreads and creeps (Rus. *stelitsya*), it grows in all conditions, it just grows like… Look, there is a word ‘*sporish*’ (in Russian: *quarrels*)—he says in a lecturing tone. – it just quarrels with nature, you can even trample on it, it is a very life-loving plant.” (Fig. 4).

“What is *sporysh* in Shiri language, then?”—Iwona asks.

“In Shiri, I don’t know”—says Abdulkadyr. His brother also shrugs his shoulders.

On another occasion, in March 2019, Rabadan (in his 50s) and Marat (in his 30s) were browsing through the booklet in Marat’s house in Izberbash.

“It is not *zveroboy*,” said Rabadan with confidence reading the heading and looking at the photograph (*zveroboy* is the Russian folk name for *Hypericum* sp.)

“No, it is *zveroboy*, they just took the photo like this”—argues Marat, making a gesture to suggest that the photo was taken from above. “These are *zveroboy* flowers, just photographed from here.”—he adds.

“It looks like a shrub here.”—says Rabadan. “And a half of it is missing”—he adds (Fig. 4). 2). He made a similar comment on *qːaˤnala čutni* (*Malva* spp. (*Malva neglecta* Wallr. and *M. pusilla* Sm.)).

*“Shulum* (*Mentha aquatica* L.)*,* is also not clear here. At this time (as in the picture), it is already too old”—said Marat turning the page. In his opinion, the illustrations of plants in the booklet should be “as they look when they are collected”, as otherwise it may be confusing.

### Text and illustration poaching, wayfaring, fact-checking

Patimat’s and Malaykat’s interaction with text and illustrations in the booklet described above is an exemplification of text wayfaring. The term wayfaring comes from Tim Ingold, who differentiated between modern traveller (travelling from one point to the other and not interested in the way itself) versus wayfarer (focused on the process of travelling, finding important things on the way, apprehending in movement) [26, 27]. Such interaction was specific for interlocutors whose knowledge was based on experience and passed from generation to generation—that is, (for the most part) for people who lived in Shiri (or visited often) and actively collected plants, either during forest-walks, or when going to the pastures. It is mostly women who belong to this group; however, men living in Shiri usually also possess knowledge about plants, particularly those they collected for snacks in childhood. People who have a vast amount of embodied knowledge, plus practice in plant collection, tend to interact with the illustrations physically (by touching, tracing the contour of the drawing with the finger). They behaved likewise with pressed herbarium specimens (explained later). Through touching, tracking and remembering the contour, they check if the plant is as named above. Bodily experience of plants is so crucial for them that contour tracking proves to be the best way to recognize the plant in the illustration. Such a way of deciphering the illustration is not necessarily obvious for a reader more familiar with botanical illustration; looking for details relying solely on vision would be much more intuitive for an Ingoldian “modern reader” [27]. Touching the illustrations resembles the idea proposed by the authors mentioned below: to combine poking (here poking the illustrations and herbarium specimens) with de Certeau’s [22] poaching (Matsutake Research Group [28] and then Eben Kirksey, Craig Schuetze, and Nick Shapiro [29]). The above researchers observed the affinity of the English word “poach” with the French word *pocher* (to push or poke with a finger or pointed instrument, to pierce). Kirksey uses the following metaphor: “At the Multispecies Salon, panelists poached each other’s papers, like chefs “poach” pears, using red wine and honey to intensify and transform the flavour of the fruit.” [29]. Similarly, Patimat and Malaykat “poach” the contours of drawing to intensify the experience, to make it more similar to their everyday practice, and to be able to recognize the plants they collect and use on a daily basis.

Fatima did not recognize *žibžni* (*Polygonum aviculare* agg.), a species she knows and collects for *ħuˤlkni* (a pie with filling, also referred to as *chudu*). The illustration she was watching is a botanical print, showing the plant abstracted from the environment (Fig. 5). That is why it looks like it has shoots pointing upwards. *Polygonum aviculare* agg. is a plant species (or rather, an aggregate species) presenting in natural environment a high diversity of shapes, depending on the habitat it grows in (bigger, more numerous leaves and an upright shape in fertile habitats; creeping habit and smaller leaves in places with less available soil and high trampling levels). The two-dimensional, de-contextualized picture did not enable her to identify a plant she knows very well and easily recognizes in various habitats (for example, in the Shiri Village centre, where it grows in poor soil and is prone to tramping by animals and people). Fatima was quite sure that the illustration is not proper.

Shiri people were proud to possess such a booklet; they showed it to their neighbors from different ethnic groups who eagerly invited us to their regions to prepare a similar booklet for them. It surprised us, however, that neither Shiri men nor women expressed a need for information about local uses, or local recipes to be included. This information, when printed, seemed irrelevant, as if from a different knowledge order. Other parts of the plant description (if read at all) were not questioned—on the contrary, they served as a confirmation of one’s knowledge—“look, it’s written here.” Some of our interlocutors hoped that the book would reveal plants in Shiri that have valuable medical properties, employable to cure such illnesses as cancer. It turns out that although they were mostly interested in medical properties and occurrence in other parts of the world (or the former USSR), they never actually read it (we do not know, however, if they would actually apply it if somebody was in medical need). This knowledge was, therefore, meant rather as pure information, to be collected but not practised 11. The authority of the rigid botanical text seemed prevailing.

As we can see from the case studies, people do a lot of textual poaching^12^ [22]. They select the information needed, and change it according to their goals. Our interlocutors freely inscribe meanings to the texts they are reading, poaching into them or, as de Certeau puts it, they “convert the text through reading and to ‘run it’ the way one runs traffic lights” ([22] p. 176). Their practice of reading rarely—or even never—resembles the practice de Certeau ascribed to “elite literati” [22]. “Elite literati” claim the rights to inscribe “proper” meanings to texts, and assume that people reading these texts internalize those meanings. In a way, researchers who fear the influence of books on LEK behave as such “elite literati.” De Certeau’s theory refers to the written texts; the case studies presented below, will show that this remark may be as well extended to the way people interact with illustrations.

Adbulkadyr’s interaction with text and illustrations is an exemplification of an interaction that we called fact-checking. He engaged mostly with the text, not the illustrations, and he was the first to find spelling mistakes in the table of contents and in particular entries. He partly read the descriptions, in particular the sections about medical characteristics. He also paid attention to the Russian names, both folk and official (he also kept a notebook full of Shiri plant names translated into Russian). The “fact-checking” kind of interaction between individuals and text is more discursive—the authority of the written text matters most. The written names are linked to the general knowledge, but not necessarily to the practice of plant collection. Abdulkadyr also paid close attention to the etymology of plants’ names—a fact possibly related to the emphasis put on it both now and in the Soviet times, especially in linguistics and literature studies (see for example [30, 31]). He remarked that *mat'-i-machekha* (*Tussilago farfara* L.) means *mat'* (mother) and *machekha* (step-mother), and *zveroboy* is an animal killer (cf. [32]). On other occasions, he also asked his wife about the meaning of a particular plant name, which seemed more important to him than the plant itself. It has to be mentioned here that Abdulkadyr had also been working on a book about Shiri and collected Shiri plants names, assigning Russian plant names to them rather than to particular species (a practice noticed by Łuczaj in regard to some Polish ethnographers [33, 34]). Generally, the knowledge about plant names and their meanings seems to be more discursive than practical knowledge—*mat'-i-machekha* is not widely used, while *zveroboy* is collected but rarely actually used (also, neither of the plants has a local name). Nevertheless, they are often mentioned when the conversation is conducted in Russian: both plants are characteristic for the Russian pharmacopoeia, and popular in media the discourse on plants. Such interaction was specific to individuals with LEK characterized by high interest in plants, but based on a discursive rather than practical knowledge. They left Shiri in childhood, or early adulthood, and have little experience of plant collection. They may, however, have subscribed to magazines, or read Soviet-style botanical books^13^. They generally valued the authority of the written text. As otherwise “knowledgeable” people (teachers, professors), they usually held high positions in their *tukhums* (lineages) and were asked for advice.

Rabadan and Marat’s interaction with the text and illustrations is an exemplification of an interaction that we called “between wayfaring and fact checking.” Sõukand, referring to Ingold, showed that if people do not engage with books, they are always wayfaring, and if they use books, they behave like the modern traveller [9]. Kołodziejska shows that it is strictly related to the type of plant they are looking for and the purpose of the walk [10, 35]. Similar observations can be made for text. Both Rabadan and Marat had collected plants while living in Shiri in their childhoods, but since then they had little actual experience with plant collection: they remembered the plants the way they looked during the time of collections, but had little memory of them in other seasons. These kinds of interactions were specific to people who grew up in Shiri and moved out to the lowlands as adults or young adults. In childhood, they actively collected plants, either as snacks, or helping the grown-ups. Nowadays, they visit Shiri only occasionally (for holidays or funerals) and rarely collect plants. Their interest in plants usually does not reach the extent of subscribing to magazines or acquiring books on plants.

**Fig. 6:**
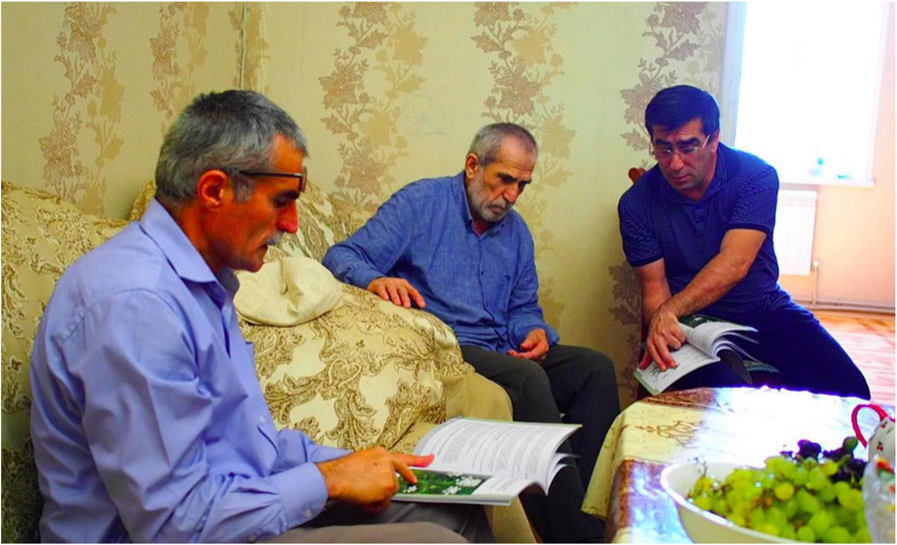
Browsing the booklet. Brothers and father discussing all entries in the booklet. The photo was taken in August 2017 in Makhachkala, Daghestan, Russian Federation. Author: Iwona Kaliszewska

For a number of our interlocutors (including Rabadan and Marat), some kind of confusion was caused by the difference between the phenological phase in which the plant is collected by the Shiri people, and the phenological phase it was presented in the illustration. The local people would rather have the illustrations showing the plant at the time when it is collected. The idea that showing photographs with different phenological phases, which is often done to facilitate plant recognition, and in consequence elucidation of as many plant species collected by the surveyed community as possible, did not correspond with the ideas of our interlocutors, who wanted illustration to represent the moment of collection (cf. [36]). Also, they perceived the plant strictly as a whole, and the parts displayed on the figure (or figures, containing separately the root, the main part, and the flower, a practice quite typical for botanical prints) were confusing them. The criticism of graphics and photographs of “too old” or “too young” plants, as well as illustrations showing “useless” and dissected specimens, was present in all kinds of interactions (Fig. 6).

The types of interactions analyzed above, though more typical for particular groups of LEK-holders, may be observed across the groups, as every person in particular situation may interact with texts and illustrations in any of the three ways. Overall, the interactions with written text/illustrations of plants are influenced by the level of engagement with plants (or a lack thereof), an individual’s gender, and formal education, as well as his or her attitude towards the written text and its authority.

### Methodological implications

Our research enabled us to notice three methodological implications, the first one concerning ethnobotanical methods which rely on showing photographs and pressed specimens, the second one concerning gender, and the third one, video-recording.

In ethnobotanical manuals (e.g., [37, 38]) one of the methods of collecting and verifying ethnobotanical data advises researchers to show local people illustrations of plants and ask them if they know or collect them. As illustrated in our case studies, pictures were only considered as proper by our interlocutors when they featured the plant in the vegetational phase when it is collected, when the parts of the plant they would use were clearly depicted, and when they were the “right” size, that is 1:1 proportion, not a close up. Thomas et al. similarly argue for 1:1 depictions but while they used magnified photographs of plant parts to facilitate species identification, in our case such depictions were confusing for informants [36]. Therefore, using illustrations for the purpose of facilitating research is not always an auspicious idea. Such properties of photographs as the picture angle, magnification, which parts of the plant specimen are presented, as well as the specimen chosen for the photo, for best field performance should be consulted with community members. It is important to remember that although the perception of illustrations is culture bounded, it also is to some extent individual. Researchers working in communities without intense contact with written texts and illustrations, for example, Reyes-Garcia and her team for Tsimané in Bolivia, demonstrated that people had problems connecting an illustration of a plant with the actual species [39]. Kołodziejska has shown that in Ukraine even those who had experience in plant collection and book reading had often great difficulties with this task [35].

Another methodological implication of our research concerns methodology applied by some scholars [36] and advised in classical ethnobotanical research manuals ([37, 38]). It is based on showing pressed herbaria specimens to local people. Our research revealed that interaction with illustrations was similar to interaction with pressed herbaria specimens. For example Patimat, who has a vast experience in plant collection, took a drying *ʡaˤʁʷamura* (*Cerastium davuricum* Fisch. ex Spreng.) specimen from our herbaria collection and traced the contour of the plant with her finger. Then (to our dismay) she took its leaf in between her fingers and started rubbing it, asking: “What is this?” “Did we collect it? Hmm.” *Cerastium davuricum* Fisch. ex Spreng*.* was among the most important plants collected in Shiri, it was considered unique. That is why we initially thought that we collected the wrong specimen, but *ʡaˤʁʷamura* collected in the following days also turned out to be *Cerastium davuricum* Fisch. ex Spreng. Shiri people with less experience had even more trouble recognizing pressed herbaria specimens (cf. [12]). In the research of Thomas et al., the recognition of voucher specimens was around 77%, and of photographed plants 94% [36].

Once plants are no longer three-dimensional, taken out of their natural habitat, people touch them, rub them, or bend them. Through this interaction, through touch, they give them another dimension (cf. [36]). Our interlocutors, however, did not have a problem with identifying three-dimensional dried plants they had dried themselves (usually by hanging bundles of herbs on a line), or the ones that were dried by their neighbors. Here, some comparison can be made to Ingold, who differentiated between the modern text and the manuscripts written by scribes in the Middle Ages [40]. He writes: “But the lines of the plot are not traced by the reader as he moves through the text. They are rather supposed to be laid out already before the journey begins. These lines are connectors. To read them, as Leroi-Gourhan realized, is to study a plan rather than to follow a trail. Unlike his medieval predecessor – an inhabitant of the page myopically entangled in its inky traces – the modern reader surveys the page as if from a great height. Routing across it from point to point (...)” [40].

Another methodological implication concerns the gender of interlocutors and researchers. In most cases, the most knowledgeable individuals in regard to plants were the women. However, in the presence of men, out of respect they did not demonstrate their plant knowledge unless explicitly asked by men or by us. Men often consulted them in regard to plant names. Men watching the booklets were more self-assured and ready to contest the female researchers’ authority^14^, whereas women were more reserved and only occasionally dropped a comment. Also, men paid more attention to plants they had collected in childhood (snacks) and plants that are easy to depict, but they skipped many other species. Therefore, researcher should be aware of such gender-induced implications (for example, [15]), in regard to the interlocutors as well as herself/himself. Other elements of researcher’s positionality, such as the country of origin or individual knowledge, should also be considered. In our case the latter was not a significant factor, as in that part of the fieldwork we were predominantly interested in the perception of booklets rather than our interlocutor’s plant knowledge (cf. [41]). Coming from a post-socialist country and speaking fluent Russian rendered us “less foreign.” Also, we did not receive the status of “knowledgeable foreign professors,” which is a quite common turn of events for Western researchers in the post-colonial world. This granted us a convenient and useful position of “students” who need to be taken care of, and have the world explained to them. In ethnobotanical research, local assistants are often hired to help with the research and translation—in our case such help was not necessary because we did not want to elicit specific knowledge, but rather focus on interaction with the text and illustrations.

The last methodological implication concerns video-recording. We realized that methodology based on video-recording was indispensable for this research to be successful. It enabled us to analyze details of bodily interactions with booklets which could otherwise be omitted. This kind of methodology is, however, not possible without long-term stays and great levels of trust between the interlocutors and the researcher.

## Conclusions

In ethnobotanical research, it is important to pay more attention to people’s interactions with their sources of knowledge, including text and illustrations. Without scrutinizing these interactions, it is hard to fully understand the influence of sources of knowledge on people’s LEK. What is more, ignoring parts of the social context may lead to misunderstanding of the processes of knowledge-making. This is why we shall not reject written sources of knowledge, because only by using them we can acquire a better understanding of what LEK is.

Ethnobotanists, as researchers, function in a particular context, and are embedded in discourses oriented towards bio-cultural-diversity conservation, and value heritage as such [1]. Institutional definitions of TEK and ILK (e.g., [42]) are very important as sources of agency for local communities and researchers. We do not criticize this standing point, but we do say that it is important to be aware of such positionality (cf. [43, 44]). In our opinion, what makes knowledge “traditional” is not the expertise about particular specimens or facts about them, neither is it the exact source of this knowledge, but rather the fact that this knowledge is made and re-made, and that it is important for the community. The *emic* perception of traditionality of knowledge is crucial for us.

Written text (as observed in the example of interaction with booklets and narratives about them) influences people’s discursive knowledge, but does not influence praxis of plant collection and usage. Shiri people were more interested in just having booklets than reading/using them, so the influence of the booklet on their plant knowledge was effectively negligible. Although they wanted to know the medicinal properties from the official pharmacopoeia, and the uses in other parts of the world (or the former USSR), this knowledge was rather regarded as purely informational—to be collected, but not practised.

Methodology that relies on showing pressed specimens or photographs to interlocutors (although in many research situations convenient and rewarding (e.g., [36, 45])), may be a very misleading way of collecting ethnobotanical data. Also, the gender of the interlocutors and researchers has to be taken into account. For further research, we suggest that it would be beneficial to conduct comparative and broader studies of the interactions between people and text and plant illustrations in various communities.

## Data Availability

Audio and video recordings are stored on external disks belonging to the Institute of Ethnology and Cultural Anthropology, University of Warsaw held by the first author – Iwona Kaliszewska. The voucher specimens are stored in the University of Warsaw Botanic Garden herbarium.

## Footnotes

1 We chose to use the term LEK (Local Environmental Knowledge) which is less prone to essentialization since the definitions of TEK (Traditional Environmental Knowledge) as well as ILK (Indigenous Local Knowledge) are also defined in legal terms which makes them political and connects them more strongly to heritage discourses which may be helpful to save the heritage but at the same time, it reifies the local community and its knowledge (cf. [1]). It is, however, important to emphasize that “knowledge” here is seen as dynamic and processual (cf. [2]).

2 Documentation of grammar, dictionary and use of Shiri languages is one of the goals of the project: “Documenting Dargi Languages in Daghestan: Shiri and Sanzhi” financed by the Volkswagen Foundation. Some results can be viewed on the project’s website [13].

3 Compare Nicolette Makovicky on lace-making knowledge. She shows that what people from the researched community see as their traditional knowledge is equally important to what researchers or politicians say about TEK [20].

4 Authors' experience of fieldwork in Daghestan, 2004–2019 as well as directly among the Shiri community between 2012–2019, was useful for a broader cultural context.

5 Plant and author names were verified according to The International Plant Name Index [23] and The Plant List [24]. Local Shiri names were transcribed according to the standard transcription for Caucasian languages. The research was done in accordance with the ISE and AAA codes of ethics, where appropriate. Verbal informed consent was collected from all of the participants of the research.

6 The booklets were part of the community materials prepared during the "Documenting Dargi languages in Daghestan: Shiri and Sanzhi" financed by the Volkswagen Foundation.

7 Both researchers speak fluent Russian.

8 Insertions in Shiri language during interviews were later translated by our interlocutors or linguists who were simultaneously working on the documentation of Shiri language.

9 Abdulkadyr had been long involved in our project, helping co-create the booklet. He is a lecturer at the Islamic and technical university in Makhachkala and is regarded by Shiri people as a knowledgeable man.

10 Rus. *polnostyu ochisshchaet organism.*

11 Similarly Makovicky shows that lace-patterns collected by lace-makers in Banska-Bystrica in Slovakia were often meant to be collected rather than applied [20].

12 We use this term after de Certeau, as a neutral term, without its negative connotations.

13 On the influence of books and magazines on LEK in Ukraine see [35] (an unpublished PhD thesis).

14 Male linguist researcher who was present during some parts of this research was listened to more attentively and was rarely interrupted.
